# On the Use of Ioduret of Potassium in Syphiltic Affections

**Published:** 1845-10

**Authors:** 


### PRACTICAL MEDICINE, &c.

*On the Use of Ioduret of Potassium in Syphiltic Affections.*—The report, which M. Gauthier has recently published respecting the curative power of this salt of Iodine, in secondary and tertiary syphiltic affections, is, on the whole, highly favorable to its use. He has administered it in a vast number of cases, and has rarely noticed any injurious or even unpleasant effects fairly attributable to its operation. On a few occasions, it appeared to cause a salivation; which, however, speedily ceased. Now and then, an innocuous exanthem made its appearance on the surface. In some persons it causes slight gastric irritation; but in most, the digestive functions appear to be decidedly improved under its use. In no instance has any wasting of the body seemed to be induced by it, as has occasionally been observed with respect to Iodine. One of the most constant effects of the Ioduret is to increase the flow of the urine. It seems to pass very rapidly into this and the other secretions; its presence is readily discoverable by its well known appropriate tests. M. Gauthier has often detected it in the saliva.

The following are the forms of the syphiltic disease in which he has witnessed the most decided curative effects, Pains of the *bones*, even when most severe, are often very rapidly and effectually subdued; nay, when caries exist, a salutary change is not unfrequently obtained. Thus in Ozœna, complicated with disease of the palate or nasal bones, we seldom fail in greatly benefitting, if not in curing, the disease. In various tubercular affections of the *skin* and *mucous membranes*, the Ioduret will be found most useful. Deep ulcerations of the throat and pharynx, rhagades or fissures about the anus and nails, will not unfrequently heal up most satisfactorily, even when mercury has been previously tried and failed. It is sometimes truly marvellous to witness the decided improvement of the general health, in the course of a few days, under the use of the Ioduret, when judiciously administered. M.

Gauthier considers that it is a most valuable remedy in many cases of mercurial cachexy: an ioduretted gargle will often serve to check salivation from this cause.

He invariably begins its administration in small doses, from two to four grains, twice a day. The quantity should be doubled every third or fourth day, until it reaches 15 or 20 grains. This dose should be continued for some time; but, if it fails in producing any decided effect upon the disease, it may be increased to two scruples or even a drachm. In a few cases, he has given as much as two drachms in the course of twenty-four hours.

A solution of the Ioduret in water, to which some tincture of Iodine has been added, may be advantageously used as a gargle in ulcerated sore-throat, and as a wash to ulcers on the surface, or on the Schneiderian membrane.

The average period, during which the internal use of the Ioduret should be continued, may be stated to be from six to eight weeks. Much will depend on the gradual increase of the doses given. Many cases will remain stationary, if the quantity of the salt administered be not progressively—and this, too, rapidly—augmented.—*Medical Examiner*, from *Medico-Chirurgical Review*, from *Observations pratiques sur le Traitment des Maladies Syphiltiques par l'Iodure de Potassium, by M. L. Gauthier.*

